# Supplementary material for: Acute stress causes sex-specific changes to ventral subiculum synapses, circuitry, and anxiety-like behavior
Source: Nat Commun. 2025 Jul 1;16:5604. doi: 10.1038/s41467-025-60512-y (PMC12217864; doi:10.1038/s41467-025-60512-y)
Supplement: Supplementary file 1 — Supplementary Information [file 41467_2025_60512_MOESM1_ESM.pdf]

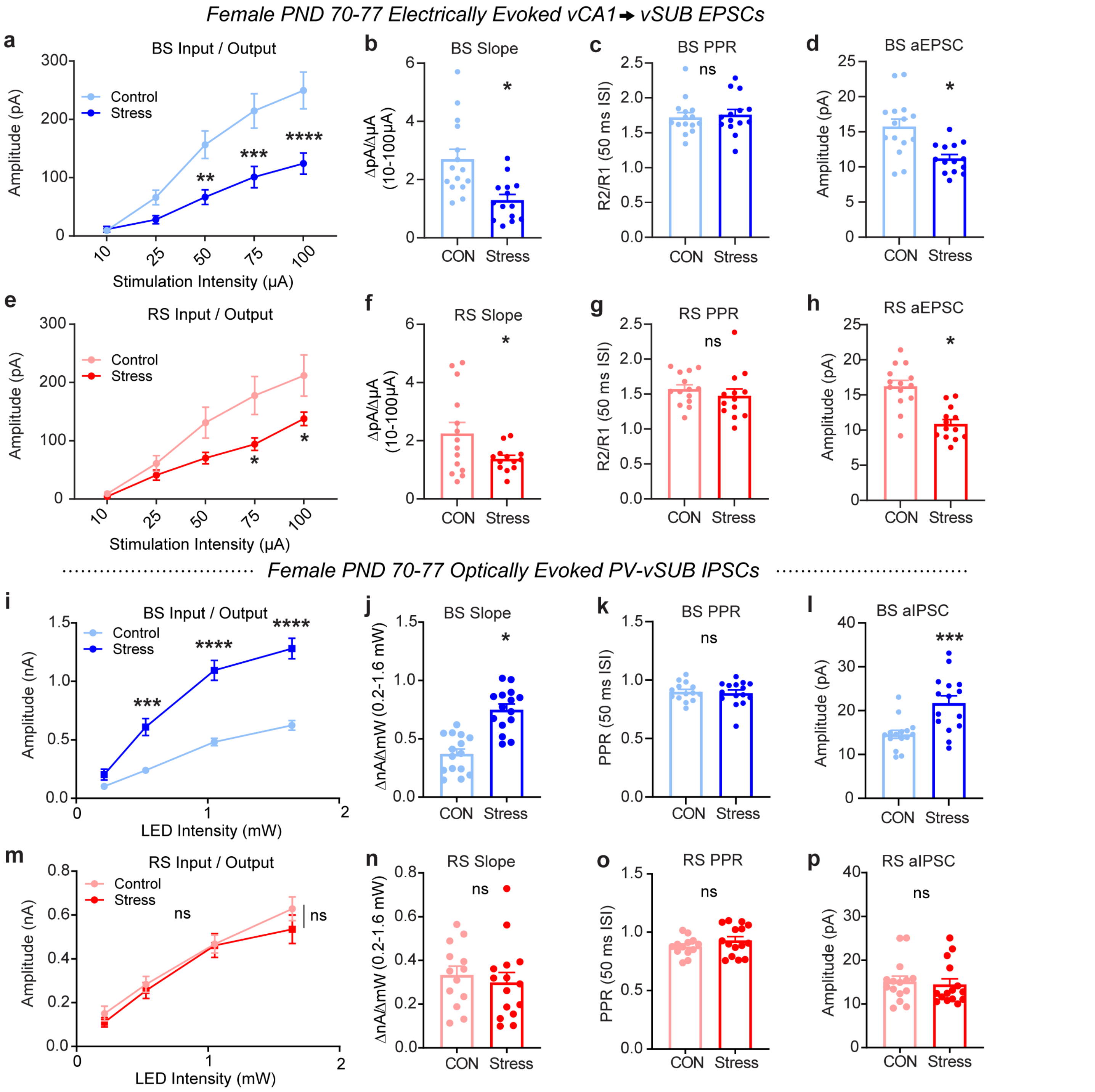

Supplemental Figure 1 Stress-induced microcircuit changes in vCA1-vSUB and parvalbumin-burst spiking (PV-BS) synapses are consistent from sexually mature adolescent (PND42-60) and young adult female mice (PND70-77). (**a**, **e**) Electrically stimulated vCA1-vSUB excitatory postsynaptic currents (EPSCs) were recorded in BS or regular spiking (RS) cells. (**a**, **e**) Input-output summary graph and (**b**, **f**) slope for EPSCs recorded in BS (**a** LED Intensity x Stress group F(4, 108)=11.49, ****p<0.0001: 10 μA, p>0.9999, 25 μA, p=0.5798, 50 μA, **p=0.0054, 75 μA, ***p=0.0002, 100 μA, ****p<0.0001; **b** slope, *p=0.0248 (t=3.506, df=4); control n=15/3, stress n=14/3) or RS cells (**e** LED Intensity x Stress group F(4, 100)=4.096, **p=0.0041: 10 μA, p>0.9999, 25 μA, p=0.9604, 50 μA, p=0.1428, 75 μA, *p<0.0157, 100 μA, *p<0.0423; **f** slope, *p=0.0471 (t=2.088, df=25); control n=14/3, stress n=13/3). (**c**, **g**) PPR measurements from BS (**c** p=0.7617 (t=0.3247, df=4), control n=15/3, stress n=14/3) and RS (**g** p=0.4052 (t=0.8467, df=25) control n=14/3, stress n=13/3). (**d**, **h**) Strontium-mediated aEPSCs after electrical stimulation in BS (**d** *p=0.0274 (t=3.393, df=4) control n=15/3, stress n=14/3) and RS (**h** *p=0.0148 (t=4.108, df=4 control n=14/3, stress n=13/3) cells. (**i**, **m**) Optically evoked inhibitory postsynaptic currents (IPSCs) were recorded in BS or RS cells. (**i**, **m**) Input-output summary graph and (**j**, **n**) slope for IPSCs recorded in BS (**i** LED Intensity x Stress group F(3, 84)=23.68, ****p<0.0001: 0.213 mW p=0.2385, 0.518 mW ***p=0.0006, 1.050 mW ****p<0.0001, 1.640 mW ****p<0.0001; **j** slope *p=0.0107 (t=4.511, df=4), control n=15/3, stress

n=15/3) or RS (**m** LED Intensity x Stress group F(3, 78)=0.6869, *p*=0.5627; **n** slope *p*=0.5809 (t=0.5591, df=26), control n=13/3, stress n=15/3). (**k**, **o**) PPR measurements from BS (**k** *p*=0.7704 (t=0.3123, df=4), control n=14/3, stress n=15/3) and RS (**o** *p*=0.2100 (t=1.285, df=26); control n=13/3, stress n=15/3). (**l**, **p**) Strontium-mediated aIPSCs in BS (**l** ****p*=0.0008 (t=3.778, df=28), control n=15/3, stress n=15/3) or RS (**p** *p*=0.7461 (t=0.3470, df=4), control n=15/3, stress n=15/3) cells. Data are represented as mean +/- SEM; means were calculated from the total number of cells. Numbers in the legend represent the numbers of cells/animals. Statistical significance was determined by a 2-way repeated measures ANOVA followed by Šidák's multiple comparisons test (**a**, **e**, **i**, **m**) or a nested unpaired Student's *t*-test (two-tailed) (**b**-**d**, **f**-**h**, **j**-**l**, **n**-**p**). Source data are provided as a Source Data file.

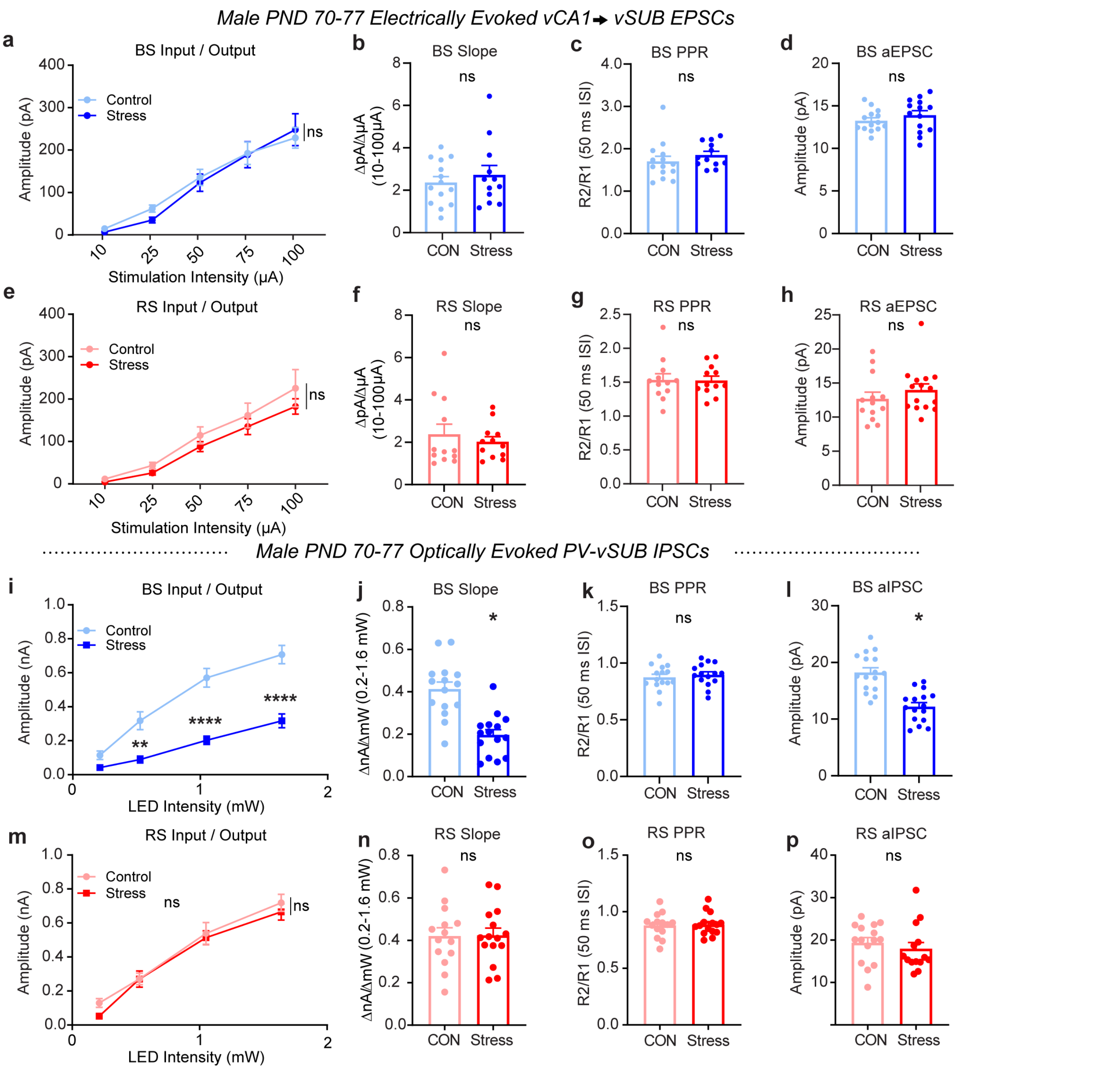

Supplemental Figure 2 Stress-induced microcircuit changes in vCA1-vSUB and parvalbumin-burst spiking (PV-BS) synapses are consistent from sexually mature adolescent (PND42-60) and young adult male mice (PND70-77). (**a**, **e**) Electrically stimulated vCA1-vSUB excitatory postsynaptic currents (EPSCs) were recorded in BS or regular spiking (RS) cells. (**a**, **e**) Input-output summary graph and (**b**, **f**) slope for EPSCs recorded in BS (**a** LED Intensity x Stress group F(4, 96)=0.6595, *p*=0.6216; **b** slope *p*=0.5639 (t=0.6283, df=4), control n=14/3, stress n=12/3) and RS (**e** LED Intensity x Stress group F(4, 88)=0.3863, *p*=0.8179; **f** slope *p*=0.5126 (t=0.6514, df=22), control n=12/3, stress n=12/3) cells. (**c**, **g**) PPR measurements from BS (**c** *p*=0.3305 (t=0.9932, df=24), control n=14/3, stress n=12/3) or RS (**g** *p*=0.9555 (t=0.05933, df=4), control n=12/3, stress n=12/3) cells. (**d**, **h**) Strontium-mediated aEPSCs after electrical stimulation in BS (**d** *p*=0.3039 (t=1.049, df=26), control n=14/3, stress n=14/3) and RS (**h** *p*=0.3246 (t=1.004, df=26), control n=13/3, stress n=15/3) cells. (**i**, **m**) Optically evoked IPSCs were recorded in BS or RS cells. (**i**, **m**) Input-output summary graph and (**j**, **n**) slope for inhibitory postsynaptic currents (IPSCs) recorded in BS (**i** LED Intensity x Stress group F(3, 84)=17.76, ****p<0.0001: 0.213 mW *p*=0.0952, 0.518 mW **p=0.0032, 1.050 mW ****p<0.0001, 1.640 mW ****p<0.0001; **j** slope *p=0.0103 (t=4.564, df=4), control n=15/3, stress n=15/3) and RS (**m** LED Intensity x Stress group F(3, 81)=0.5342, *p*=0.6602; **n** slope *p*=0.9953 (t=0.006279, df=4), control n=14/3, stress n=15/3) cells. (**k**, **o**) PPR measurements from BS (**k** *p*=0.6787 (t=0.4459, df=4), control n=15/3, stress n=15/3) and RS (**o** *p*=0.8448 (t=0.1976, df=27), control n=14/3, stress n=15/3) cells. (**l**, **p**) Strontium-mediated aIPSCs in BS (**l** *p=0.0117 (t=4.400, df=4), control n=16/3, stress n=16/3) and RS (**p** *p*=0.5250 (t=0.6956, df=4), control n=15/3, stress n=14/3) cells. Data are represented as mean +/- SEM;

means were calculated from the total number of cells. Numbers in the legend represent the numbers of cells/animals. Statistical significance was determined by a 2-way repeated measures ANOVA followed by Šidák's multiple comparisons test (**a**, **e**, **i**, **m**) or a nested unpaired Student's *t*-test (two-tailed) (**b**-**d**, **f**-**h**, **j**-**l**, **n**-**p**). Source data are provided as a Source Data file.

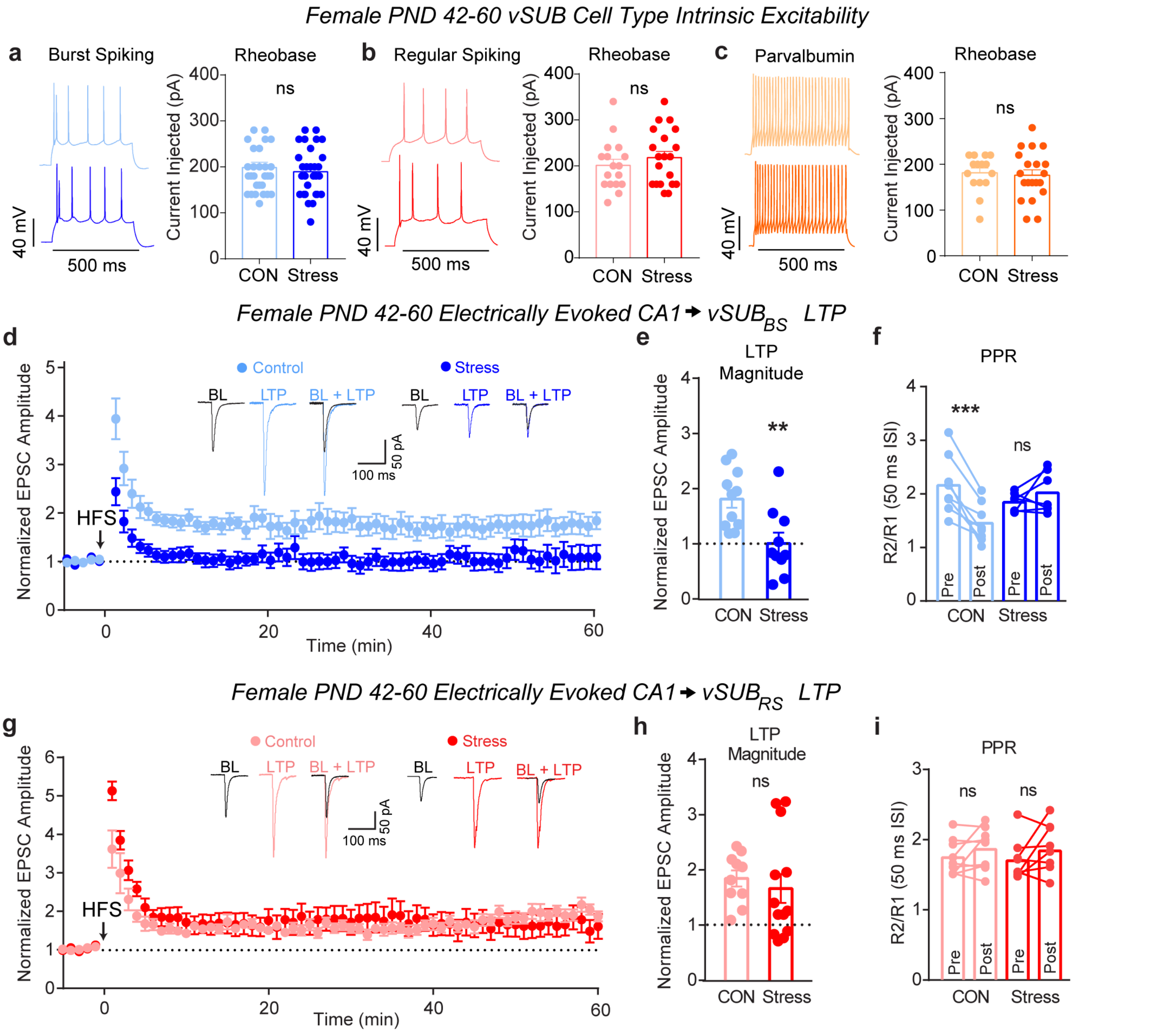

Supplemental Figure 3 Stress ablates vCA1-vSUB presynaptic long term potentiation (LTP) in burst spiking (BS) cells of females (PND42-60) without altering intrinsic excitability of vSUB cell types. (**a**) (Left) Representative responses in BS cells to +260pA and (right) the minimum amount of current required to fire BS cells (rheobase, *p*=0.8664 (t=0.1756, df=6); control n=29/3, stress n=29/5). (**b**) Representative responses in regular spiking (RS) cells to +260pA and (right) the minimum amount of current required to fire RS cells (rheobase *p*=0.3840 (t=0.8817, df=35); control n=17/3, stress n=20/4). (**c**) (Left) Representative responses in tdTomato+ parvalbumin (PV) cells to +260pA and (right) the minimum amount of current required to fire PV cells in PVAi9 mice (rheobase, *p*=0. 9165 (t=0.1102, df=5); control n=15/3, stress n=20/4). (**d**, **g**) Summary graph of LTP experiments in BS (**d**) or RS (**g**) cells with representative traces of baseline (BL) and/or LTP. (**e**, **h**) LTP magnitude from the last 10 minutes of LTP recording in BS (**e**; ***p*=0.0044 (t=3.235, df=19); control n=11/6, stress n=10/4) or RS (**h**; *p*=0.6149 (t=0.5211, df=9); control n=10/6, stress n=13/5) cells. (**f**, **i**) Pre and post LTP PPR measurements in BS (**f**; control ****p*=0.0008 (t=5.624, df=7); stress *p*=0.2826 (t=1.180, df=6); control n=8/6, stress n=7/4) or RS (**i**; control *p*=0.1970 (t=1.407, df=8); stress *p*=0.2602 (t=1.210, df=8); control n=9/6, stress n=9/5) cells. Data are represented as mean +/- SEM; means were calculated from the total number of cells. Numbers in the legend represent the numbers of cells/animals. Statistical significance was determined by a nested unpaired Student's *t*-test (**a**, **b**, **c**, **e**, **h**) or paired Student's *t*-test (two-tailed) (**f**, **i**). Source data are provided as a Source Data file.

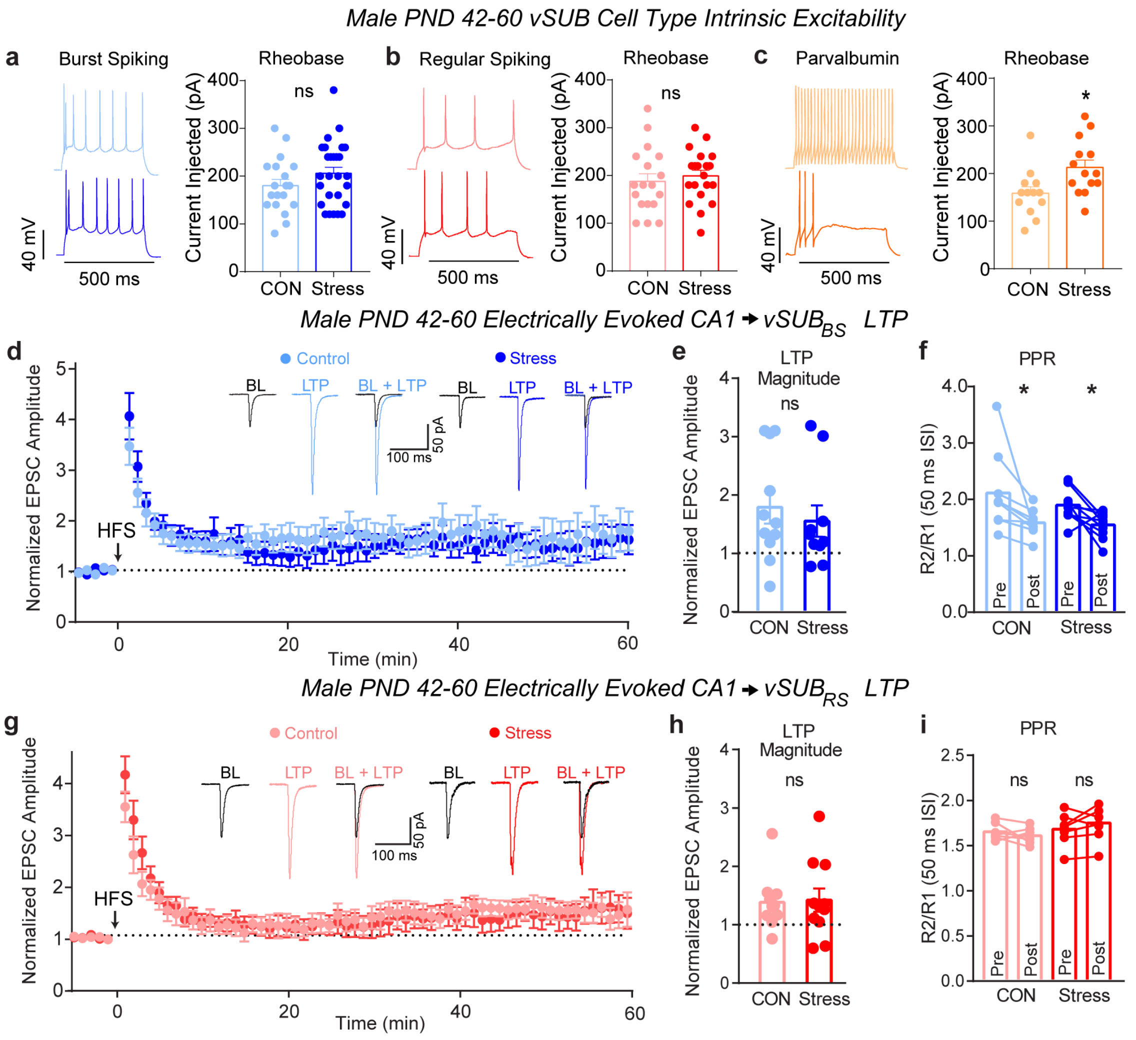

Supplemental Figure 4 Stress does not alter vCA1-vSUB long term potentiation (LTP) in males (PND42-60) and reduces the intrinsic excitability of vSUB parvalbumin (PV) cells. (**a**) (Left) Representative responses in burst spiking (BS) cells to +260pA and (right) the minimum amount of current required to fire BS cells (rheobase *p*=0.3702 (t=0.9683, df=6); control n=19/4, stress n=27/4). (**b**) (Left) Representative responses in regular spiking (RS) cells to +260pA and (right) the minimum amount of current required to fire RS cells (rheobase *p*=0.9257 (t=0.09927, df=4); control n=18/3, stress n=20/3). (**c**) (Left) Representative responses in tdTomato+ PV cells to +260pA and (right) the minimum amount of current required to fire PV cells in PVAi9 mice (rheobase **p*=0.0144 (t=2.631, df=25); control n=13/3, stress n=14/3). (**d**, **g**) Summary graph of LTP experiments in BS (**d**) or RS (**g**) cells with representative traces of baseline (BL) and/or LTP in males. (**e**, **h**) LTP magnitude from the last 10 minutes of LTP recording in BS (**e**; *p*=0.5539 (t=0.6026, df=19); control n=11/6, stress n=10/6) or RS (**h**; *p*=0.8969 (t=0.1312, df=20); control n=10/7, stress n=11/7) cells. (**f**, **i**) Pre and post LTP PPR measurements in BS (**f**; control **p*=0.0353 (t=2.528, df=8); stress **p*=0.0151 (t=2.994, df=9); control n=11/6, stress n=10/6) or RS (**i**; control *p*=0.3146 (t=1.097, df=6); stress *p*=0.1656 (t=1.548, df=7); control n=7/6, stress n=8/6) cells. Data are represented as mean +/- SEM; means were calculated from the total number of cells. Numbers in the legend represent the numbers of cells/animals. Statistical significance was determined by a nested unpaired Student's *t*-test (**a**, **b**, **c**, **e**, **h**) or paired Student's *t*-test (two-tailed) (**f**, **i**). Source data are provided as a Source Data file.

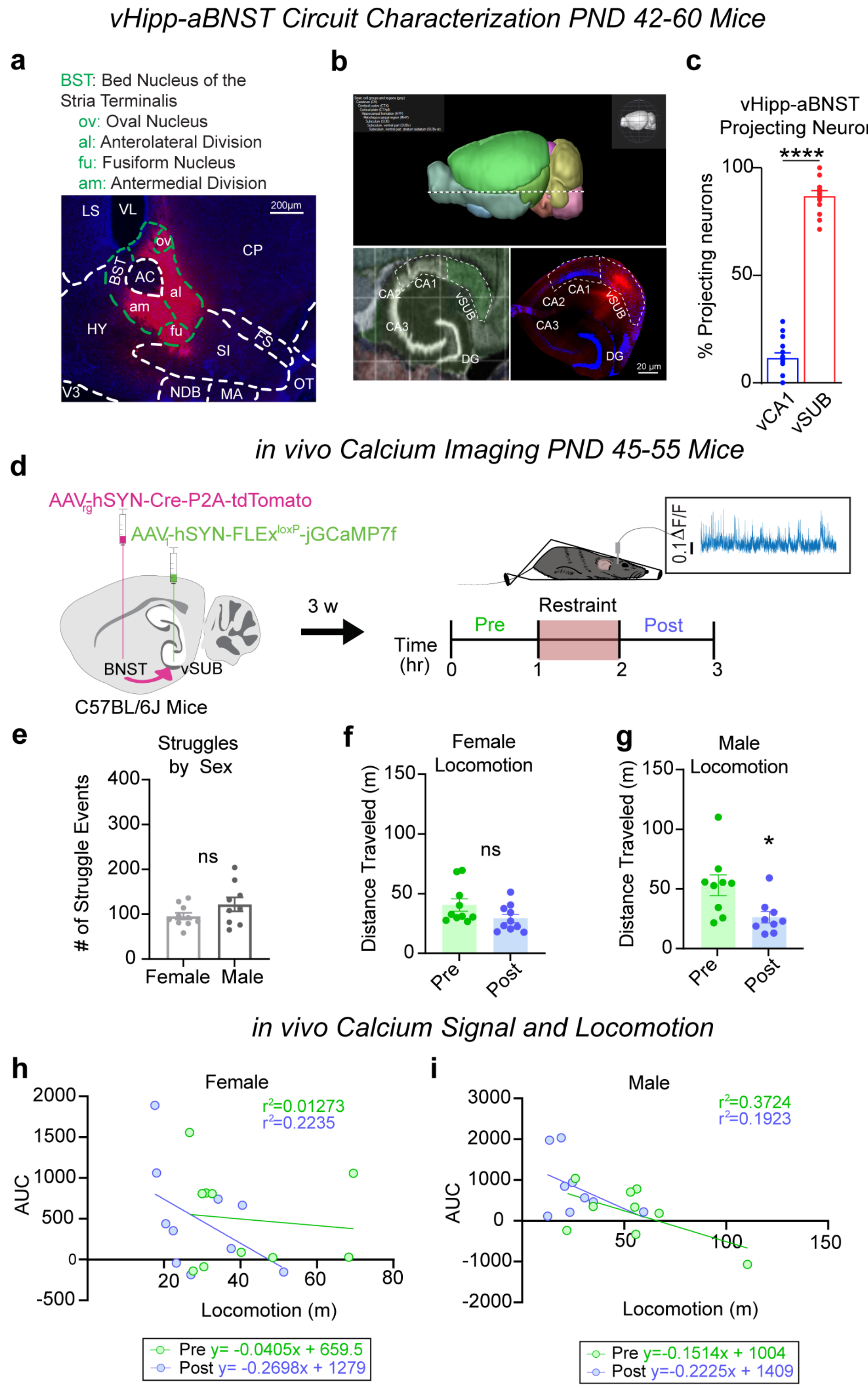

Supplemental Figure 5 Within the ventral hippocampus (vHipp), vSUB primarily projects to aBNST and locomotor activity does not correlate with vSUB-aBNST *in vivo* calcium activity. (**a**) Representative image indicating proper targeting of aBNST with retrograde AAV2mRuby. VL: lateral ventricle, LS: lateral septal nucleus, HY: hypothalamus, V3: third ventricle, NDB: diagonal band nucleus, MA: magnocellular nucleus, OT: olfactory tubercle, SI: substantia inominata, FS: fundus of striatum, CP: caudoputamen. (**b**) Diagram indicating the horizontal plane representing the vHipp sections quantified for mRuby fluorescence (top). vHipp image from the Brain Allen Explorer. Hippocampal subregions are identified based on transcriptomic profiles (Ding et al., 2020) integrated into the Allen Brain Institute's Brain Explorer. vSUB is highlighted in green (bottom left). Representative vHipp section from a similar plane as the Allen Brain Explorer with vCA1 and vSUB identified and outlined with white hashed lines (bottom right). (**c**) Quantification of % mRuby+ cells in vCA1 or vSUB that project to aBNST (**c**;*****p*<0.0001 (t=20.63, df=6), n=19 vHIPP 50 μm slices/4 mice (2 females, 2 males)). (**d**) Schematic of dual virus injection and monitoring *in vivo* calcium activity of aBNST projecting vSUB cells in

control (pre), stress, and stress recovery periods (post). Number of struggling events by sex (**e** *p*=0.1344 (t=1.572, df=17). Locomotor activity pre and post in females (**f**; *p*= 0.0921 (t=1.885, df=9), n=10) and males (**g**; *p=0.0109 (t=3.300, df=8), n=9). Locomotor activity does not significantly correlate with *in vivo* calcium signal in females (**h** pre slope F(1, 8)=0.1031, *p*=0.7564; post slope F(1, 8)=2.302, *p*=0.1677, n=10) nor males (**i** pre slope F(1, 7)=4.154, *p*=0.0809; post slope F(1, 7)=1.667, *p*=0.2377, n=9). Data are represented as mean +/- SEM; means were calculated from the total number of animals (**e**-**g**) or total number of vHIPP slices (**c**). Numbers in the legend represent the numbers of animals. Statistical significance was determined by a nested unpaired Student's *t*-test (two-tailed) (**c**), unpaired Student's *t*-test (two-tailed) (**e**), paired Student's *t*-test (two-tailed) (**f**, **g**), or simple linear regression analysis (**h**, **i**). Source data are provided as a Source Data file.

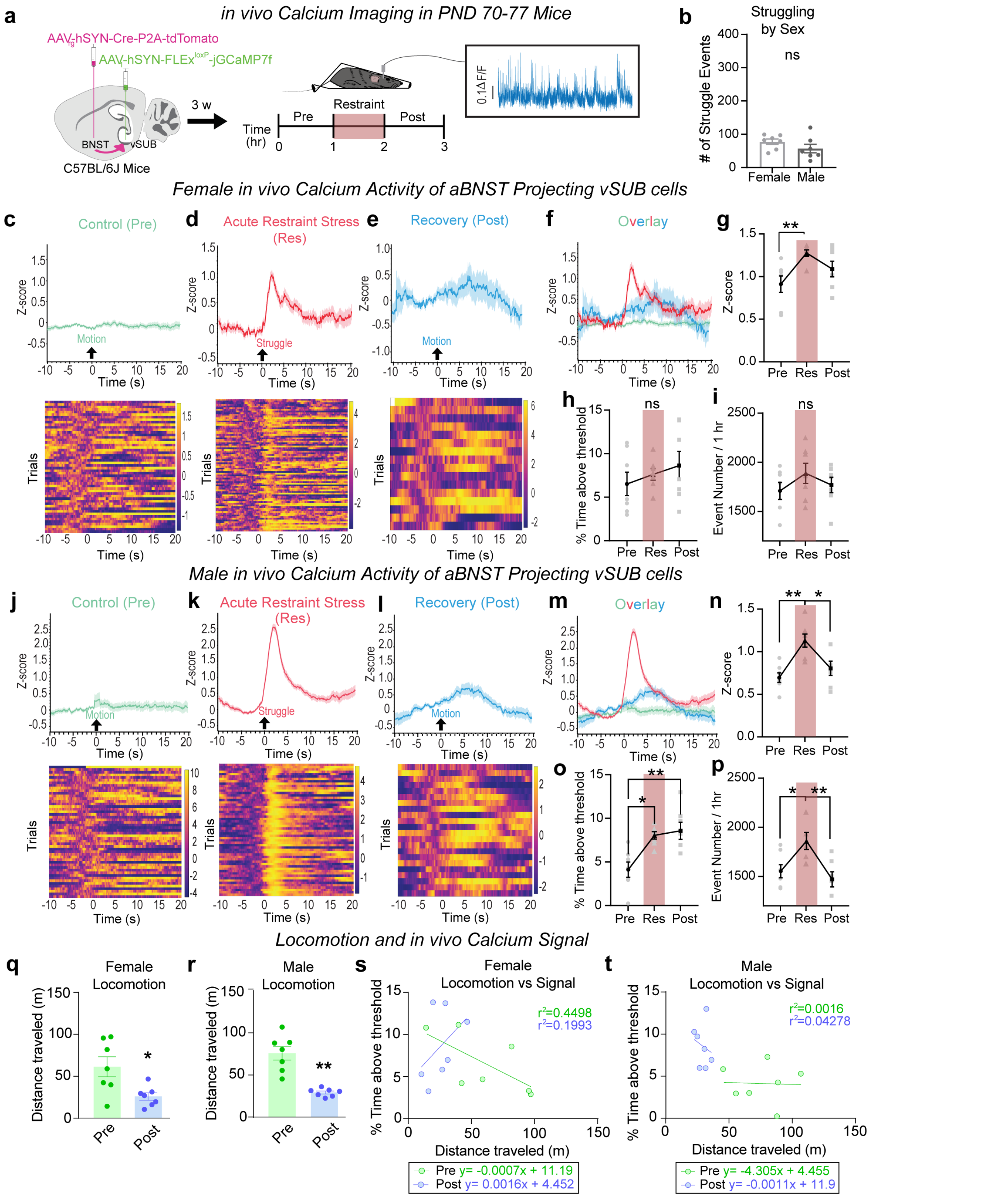

Supplemental Figure 6 Stress induces sexually dimorphic responses in vSUB-aBNST *in vivo* calcium activities in young adult (PND70-77) mice.(**a**) Schematic of dual virus injection and monitoring *in vivo* calcium activity of aBNST-projecting vSUB cells in control (pre), stress (res), and stress recovery periods (post). (**b**) Number of struggle events by sex (p=0.1997 (t=1.357, df=12). (**c-e**, **j-l**) The representative traces and heatmaps of *in vivo* calcium activities time-locked to locomotor initiation during the control phase (**c** female; **j** male) and the stress recovery phase (**e** female; **l** male) or time-locked to struggle initiation (i.e. trunk movement) during the stress procedure (**d** female; **k** male) in mice. Overlay of representative traces for females (**f**; **c-e**) and males (**m**; **j-l**). (**g**, **n**) The amplitude of calcium events represented as the Z-score of calcium events in female (**g** F(2, 12)=6.460; *p=0.0125, Pre vs Res **p=0.0095; Pre vs Post p=0.2283; Res vs

Post p=0.1968; n=7) and male (**n** F(2, 12)=9.819, ***p*=0.0030; Pre vs Res ***p*=0.0029; Pre vs Post *p*=0.5494; Res vs Post **p*=0.0198; n=7) mice. (**h**, **o**) Overall population activity represented as the percent time above threshold in females (**h**; F(2, 12)=0.6835, *p*=5235; n=7) and males (**o** F(2, 12)=9.182, ***p*=0.0038; Pre vs Res **p*=0.0121; Pre vs Post ***p*=0.0053; Res vs Post *p*=0.8916; n=7). (**i**, **p**) The average calcium event frequency during each phase in females (**i** F(2, 12)=2.532, *p*=0.1210; n=7) and males (**p** F(2, 12)=9.330, ***p*=0.0036; Pre vs Res **p*=0.0184; Pre vs Post *p*=0.6673; Res vs Post ***p*=0.0039; n=7). Locomotor activity pre and post in females (**q** **p*=0.0148 (t=3.384, df=6), n=7) and males (**r** ***p*=0.0010 (t=5.938, df=6), n=7). Locomotor activity does not significantly correlate with *in vivo* calcium signal in females (**s** pre slope F(1, 5)=4.087, *p*=0.0992; post slope F(1, 5)=1.245, *p*=0.3153, n=7) nor males (**t** pre slope F(1, 5)=0.007957, *p*=0.9224; post slope F(1, 5)=0.2234, *p*=0.6563, n=7). Data are represented as mean +/- SEM; means were calculated from the total number of animals. Numbers in the legend represent the numbers of animals. Statistical significance was determined by an unpaired Student's *t*-test (two-tailed) (**b**), 1-way repeated measures ANOVA followed by Tukey's multiple comparisons test (**g**-**i**, **m**-**p**), paired Student's *t*-test (two-tailed) (**q**, **r**), or simple linear regression analysis (**s**, **t**). Source data are provided as a Source Data file.

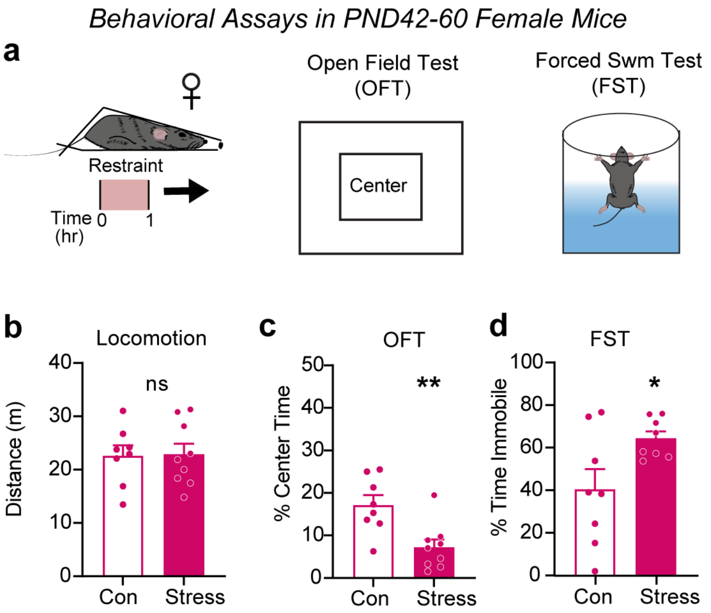

Supplemental Figure 7 Stress increases anxiety- and depression-like behaviors in female mice. (**a**) Female mice (PND42-60) were assessed for anxiety-like behaviors in the open field test (OFT) or depression-like behaviors in the forced swim test (FST) after 1-hr of acute restraint stress. (**b**) Locomotion in the OFT. (**c**) Percent time spent in the middle 1/3 of the OFT box (**c** ***p*=0.0039 (t=3.405, df=15); control n=8, stress n=9). (**d**) Percent time spent immobile during the FST (**d** **p*=0.0320 (t=2.381, df=14), control n=8, stress n=8). Data are represented as mean +/- SEM; means were calculated from the total number of animals. Numbers in the legend represent the numbers of animals. Statistical significance was determined by an unpaired Student's *t*-test (two-tailed) (**b-d**). Source data are provided as a Source Data file.

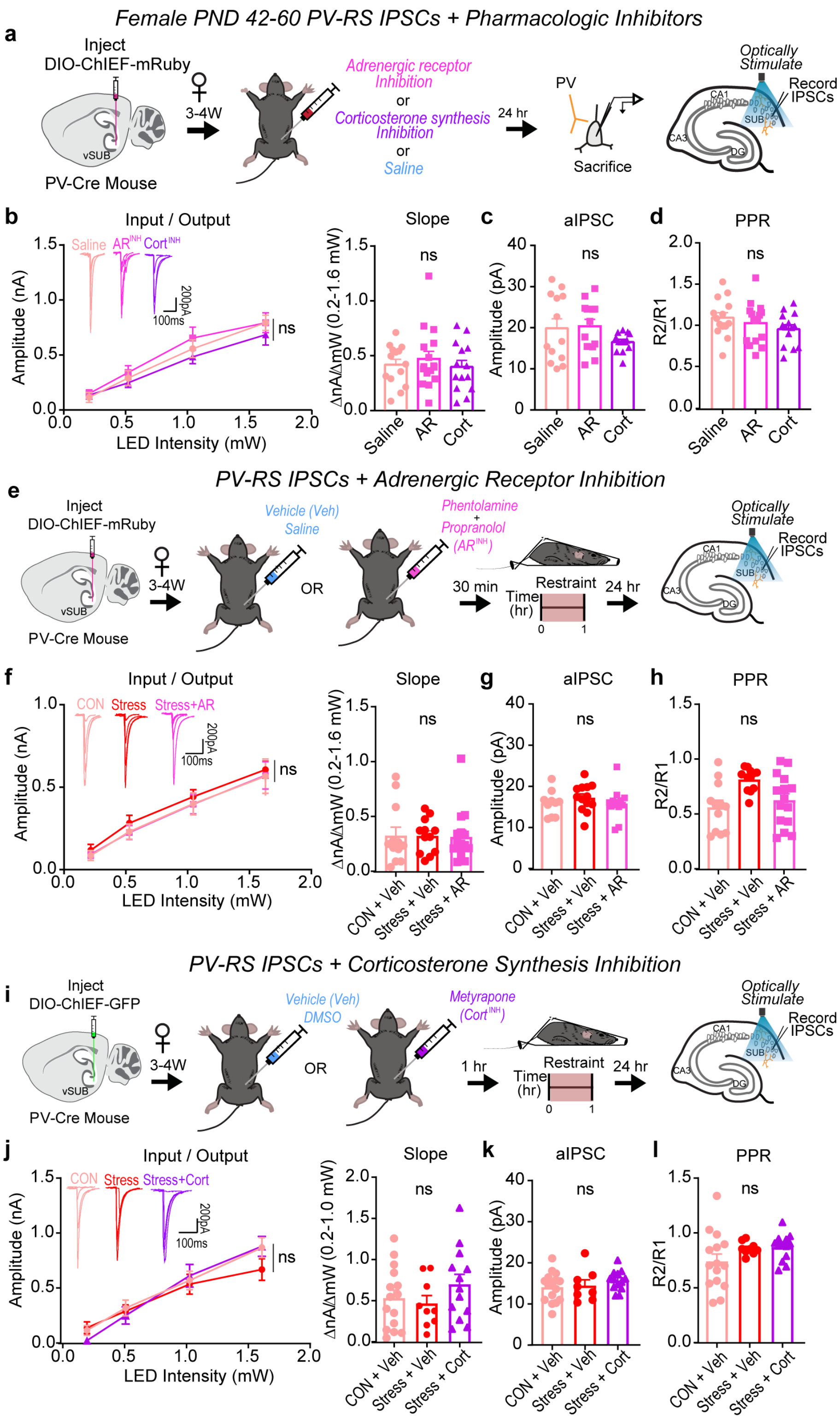

Supplemental Figure 8 Pharmacologic manipulation of adrenergic or corticosterone signaling does not impact parvalbumin-regular spiking (PV-RS) inhibition in vSUB of females. (**a**) A Cre-dependent ChIEF AVV was injected into the vSUB of PV-Cre female mice (PND42-60) and optogenetically evoked inhibitory postsynaptic currents (IPSCs) from PV interneurons were recorded from RS cells 24-hr after saline, adrenergic receptor inhibition (AR), or corticosterone synthesis inhibition (Cort) pretreatment. (**b**) Input-output summary graph with representative traces (left) and slope (right) for IPSCs recorded in RS cells after drug controls (**b**; LED Intensity x Drug $F(6, 123)=0.7989$, $p=0.5725$; slope, $F(2, 41)=0.4020$, $p=0.6716$; saline n=14/3, AR n=16/3, Cort n=14/3). (**c**) Strontium-mediated aIPSC amplitudes after optogenetic stimulation for RS cells (**c**; $F(2, 6)=0.777$, $p= 0.5008$; saline n=13/3, AR n=13/3, Cort n=13/3). (**d**) PPR (50ms) measurements from RS cells (**d**; $F(2, 6)=0.6249$, $p=0.5669$; saline n=14/3, AR n=16/3, Cort n=14/3). (**e**, **i**) A Cre-

dependent ChIEF AVV was injected into the vSUB of PV-Cre female mice, mice received a AR/saline (**e**) or Cort/DMSO (**i**) pretreatment before stress or control conditions, and optogenetically evoked IPSCs from PV interneurons were recorded from RS cells 24-hr later. (**f**, **j**) Input-output summary graph with representative traces (left) and slope (right) for IPSCs in RS cells in AR (**f**; LED Intensity x Condition $F(6, 108)=0.0226$, $p>0.9999$; slope, $F(2, 7)=0.01179$, $p=0.9140$; Control+Saline n=12/3, Stress+Saline n=12/3, Stress+AR=15/4) or Cort (**j**; LED Intensity x Condition $F(6, 102)=1.486$, $p=0.1904$; slope, $F(2, 34)=1.140$, $p=0.3319$; Control+DMSO n=15/4, Stress+DMSO n=9/2, Cort+DMSO n=13/3) studies. (**g**, **k**) Strontium-mediated aIPSC amplitudes from RS cells after AR (**g**; $F(2, 7)=0.6753$, $p=0.5393$; Control+Saline n=10/3, Stress+Saline n=13/3, Stress+AR n=13/4) or Cort (**k**; $F(2, 6)=0.8515$, $p=0.4726$, Control+DMSO n=16/4, Stress+DMSO n=8/2, Cort+DMSO n=13/3) studies. (**h**, **l**) PPR (50ms) measurements in RS cells in AR (**h**; $F(2, 7)=2.056$, $p=0.1984$, Control+Saline n=12/3, Stress+Saline n=12/3, Stress+AR n=15/3) or cort (**l**; $F(2, 6)=1.363$, $p=0.3251$, Control+DMSO n=15/4, Stress+DMSO n=9/2, Cort+DMSO n=13/3) studies. Data are represented as mean +/- SEM; means were calculated from the total number of cells. Numbers in the legend represent the numbers of cells/animals. Statistical significance was determined by a 2-way repeated measures ANOVA (**b** (left), **f** (left), **j** (left)) or nested 1-way ANOVA followed by Šidák's multiple comparisons test (**b** (right)-**d**, **f** (right)-**h**, **j** (right)-**l**). Source data are provided as a Source Data file.

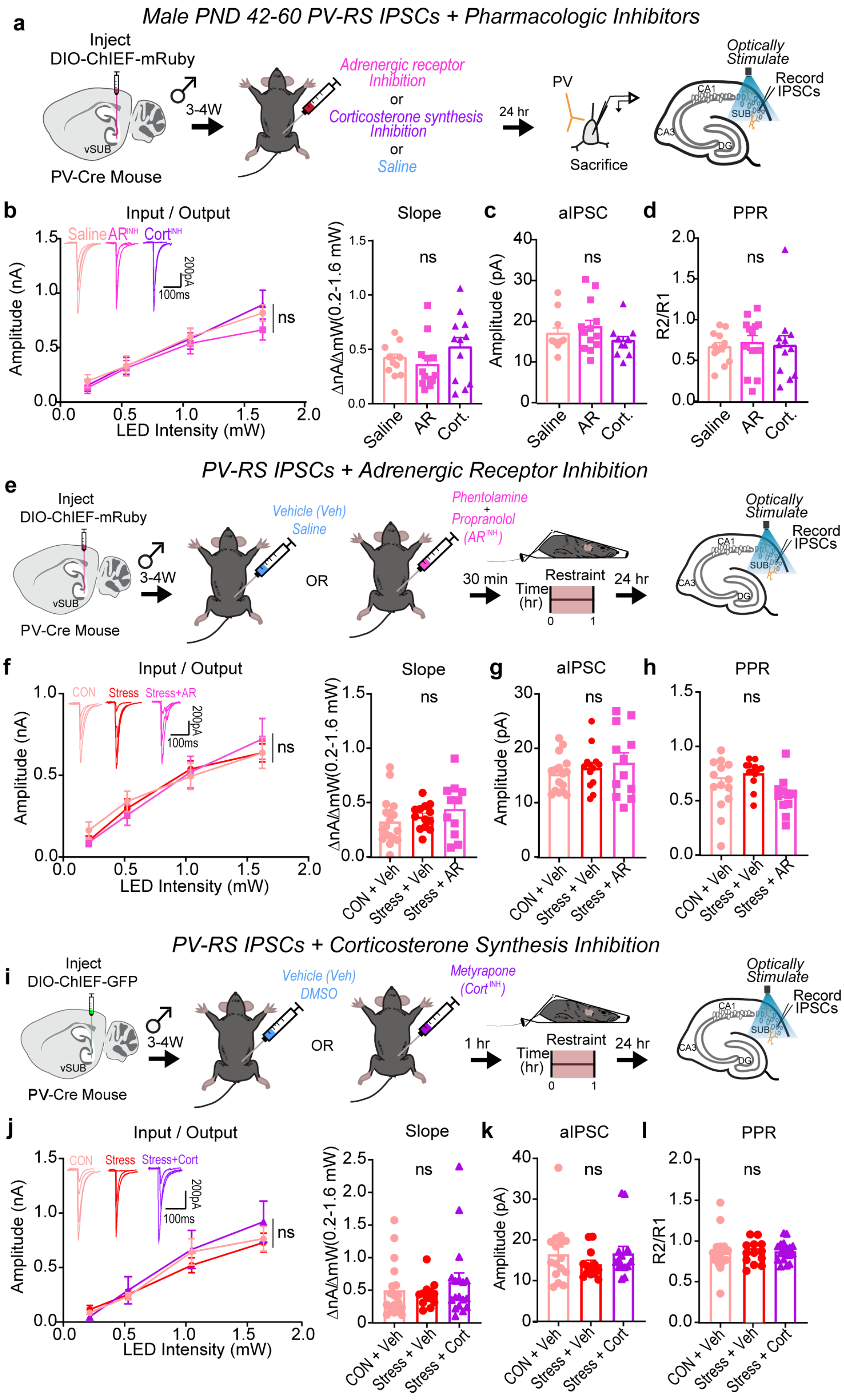

Supplemental Figure 9 Pharmacologic manipulation of adrenergic or corticosterone signaling does not impact parvalbumin-regular spiking (PV-RS) inhibition in vSUB males. (**a**) A Cre-dependent ChIEF AVV was injected into the vSUB of PV-Cre male mice (PND42-60) and optogenetically evoked inhibitory postsynaptic currents (IPSCs) from PV interneurons were recorded from RS cells 24-hr after saline, adrenergic receptor inhibition (AR), or corticosterone synthesis inhibition (Cort) pretreatment. (**b**) Input-output summary graph with representative traces (left) and slope (right) for IPSCs recorded in RS cells after drug controls (**b**; LED Intensity x Drug $F(6, 102)=1.1013$, $p=0.4218$; slope, $F(2, 6)=0.02668$, $p=0.4804$; saline n=12/3, AR n=13/3, Cort n=12/3). (**c**) Strontium-mediated aIPSC amplitudes after optogenetic stimulation for RS cells (**c**; $F(2, 6)=0.9584$, $p=0.4353$; saline n=11/3, AR n=13/3, Cort n=12/3). (**d**) PPR (50ms) measurements from RS cells (**d**; $F(2, 6)=0.02668$, $p=0.9738$; saline n=12/3, AR n=13/3, Cort n=12/3). (**e, i**) A Cre-

dependent ChIEF AVV was injected into the vSUB of PV-Cre female mice, mice received a AR/Saline (**e**) or Cort/DMSO (**i**) pretreatment before stress or control conditions, and optogenetically evoked IPSCs from PV interneurons were recorded from RS cells 24-hr later. (**f**, **j**) Input-output summary graph with representative traces (left) and slope (right) for IPSCs in RS cells in AR (**f**; LED Intensity x Condition F(6, 117)=1.319, *p*=0.2540; slope, F(2, 7)=0.9516, *p*=0.4310; Control+Saline n=18/4, Stress+Saline n=13/3, Stress+AR=11/3) or Cort (**j**; LED Intensity x Condition F6, 138)=0.7708, *p*=0.7708; F(2, 7)=0.6696, slope, *p*=0.5429; Control+DMSO n=18/4, Stress+DMSO n=13/3, Stress+Cort n=18/3) studies. (**g**, **k**) Strontium-mediated aIPSC amplitudes from RS cells after AR (**g**; F(2, 7)=0.07520, *p*=0.9283; Control+Saline n=16/4, Stress+Saline n=13/3, Stress+AR n=12/3) or Cort (**k**; F(2, 7)=0.7334, *p*=0.5238, Control+DMSO n=16/4, Stress+DMSO n=13/3, Stress+Cort n=14/3) studies. (**h**, **l**) PPR (50ms) measurements in RS cells in AR (**h**; F(2, 7)=2.926, *p*=0.1193, Control+Saline n=18/4, Stress+Saline n=13/3, Stress+AR=11/3) or Cort (**l**; F(2, 7)=0.05156, *p*=0.9501, Control+DMSO n=18/4, Stress+DMSO n=13/3, Stress+Cort n=18/3) studies. Data are represented as mean +/- SEM; means were calculated from the total number of cells. Numbers in the legend represent the numbers of cells/animals. Statistical significance was determined by a 2-way repeated measures ANOVA (**b** (left), **f** (left), **j** (left)) or nested 1-way ANOVA followed by Šidák's multiple comparisons test (**b** (right)-**d**, **f** (right)-**h**, **j** (right)-**l**). Source data are provided as a Source Data file.
