# Supplementary material for: Acute stress causes sex-specific changes to ventral subiculum synapses, circuitry, and anxiety-like behavior
Source: Nat Commun. 2025 Jul 1;16:5604. doi: 10.1038/s41467-025-60512-y (PMC12217864; doi:10.1038/s41467-025-60512-y)
Supplement: Supplementary file 2 — Reporting Summary [file 41467_2025_60512_MOESM2_ESM.pdf]

nature portfolio

Corresponding author(s): Jason Aoto

Last updated by author(s): Apr 23, 2025

Reporting Summary

Nature Portfolio wishes to improve the reproducibility of the work that we publish. This form provides structure for consistency and transparency in reporting. For further information on Nature Portfolio policies, see our Editorial Policies and the Editorial Policy Checklist.

Statistics

For all statistical analyses, confirm that the following items are present in the figure legend, table legend, main text, or Methods section.

n/a	Confirmed	
☐	☒	The exact sample size (*n*) for each experimental group/condition, given as a discrete number and unit of measurement
☐	☒	A statement on whether measurements were taken from distinct samples or whether the same sample was measured repeatedly
☐	☒	The statistical test(s) used AND whether they are one- or two-sided<br>*Only common tests should be described solely by name; describe more complex techniques in the Methods section.*
☐	☒	A description of all covariates tested
☐	☒	A description of any assumptions or corrections, such as tests of normality and adjustment for multiple comparisons
☐	☒	A full description of the statistical parameters including central tendency (e.g. means) or other basic estimates (e.g. regression coefficient) AND variation (e.g. standard deviation) or associated estimates of uncertainty (e.g. confidence intervals)
☐	☒	For null hypothesis testing, the test statistic (e.g. *F*, *t*, *r*) with confidence intervals, effect sizes, degrees of freedom and *P* value noted<br>*Give P values as exact values whenever suitable.*
☒	☐	For Bayesian analysis, information on the choice of priors and Markov chain Monte Carlo settings
☒	☐	For hierarchical and complex designs, identification of the appropriate level for tests and full reporting of outcomes
☒	☐	Estimates of effect sizes (e.g. Cohen's *d*, Pearson's *r*), indicating how they were calculated

Our web collection on statistics for biologists contains articles on many of the points above.

Software and code

Policy information about availability of computer code

Data collection	All electrophysiology recordings were acquired using Molecular Devices Multiclamp 700B amplifier and Digidata 1440 digitizer with Axon pClamp™ 9.0 Clampex software and analyzed using Axon™ pClamp10 Clampfit software. Behavioral data was acquired with Ethovision 10 software. Fiber photometry data were collected with Synapse software, a Fluorescence Mini Cube FMC4 (Doric), and femtowatt photoreceiver (Newport).
Data analysis	Electrophysiologicy recordings were analyzed with Axon™ pClamp10 Clampfit software. All statistical analyses were performed in Prism 7 (GraphPad). Fiber photometry data was analyzed with custom MATLAB scripts (https://github.com/BeierLaboratory/FiberPhotometry) or Python3.8 GuPPy (https://github.com/LernerLab/GuPPy). Behavioral data was analyzed with Ethovision 10 software.

For manuscripts utilizing custom algorithms or software that are central to the research but not yet described in published literature, software must be made available to editors and reviewers. We strongly encourage code deposition in a community repository (e.g. GitHub). See the Nature Portfolio guidelines for submitting code & software for further information.

Data

Policy information about availability of data

All manuscripts must include a data availability statement. This statement should provide the following information, where applicable:

- Accession codes, unique identifiers, or web links for publicly available datasets
- A description of any restrictions on data availability
- For clinical datasets or third party data, please ensure that the statement adheres to our policy

All fiber photometry data have been deposited at Zenodo and is publicly available as of the date of the publication along with accession codes. Source data generated in this study are provided in the Source Data file. Raw data will be provided upon request. Any additional information required to reanalyze the data reported in this paper is available from the lead contact upon request (Jason Aoto; jason.aoto@cuanschutz.edu).

Research involving human participants, their data, or biological material

Policy information about studies with human participants or human data. See also policy information about sex, gender (identity/presentation), and sexual orientation and race, ethnicity and racism.

Reporting on sex and gender	N/A, no human data were used.
Reporting on race, ethnicity, or other socially relevant groupings	N/A, no human data were used.
Population characteristics	N/A, no human data were used.
Recruitment	N/A, no human data were used.
Ethics oversight	N/A, no human data were used.

Note that full information on the approval of the study protocol must also be provided in the manuscript.

Field-specific reporting

Please select the one below that is the best fit for your research. If you are not sure, read the appropriate sections before making your selection.

☒ Life sciences ☐ Behavioural & social sciences ☐ Ecological, evolutionary & environmental sciences

For a reference copy of the document with all sections, see nature.com/documents/nr-reporting-summary-flat.pdf

Life sciences study design

All studies must disclose on these points even when the disclosure is negative.

Sample size	For all electrophysiology experiments, at least 3 independent animals were used per sex per experimental condition. At least 6 independent animals were used in behavior or fiber photometry experiments. All statistics were calculated across cells (electrophysiology) or animals (behavior or fiber photometry). Error bars represent standard error of the mean and significance was set at P=0.05 (Student's two-tailed t-test, non-parametric Mann-Whitney, one way ANOVA, or two way ANOVA). Outliers were excluded from data - Prism "Identify Outliers" was used. No statistical methods were used to pre-determine sample sizes, but we post-hoc determined sample sizes necessary to achieve 95% power from our experimentally determined effect sizes. The number of cells represented in the main electrophysiological data meet or exceed the estimated sample sizes. The sample sizes used in our study, such as the number or neurons and animals, are consistent with previous publications (Tian et al., Neuron, 2024; Tian et al., Cell Rep, 2022; Boxer et al., Cell Reports, 2021; Boxer et al., J Neuro, 2022; Aoto et al., Cell, 2013).
Data exclusions	For electrophysiology studies, cells of low quality were excluded from the electrophysiology analysis (i.e, unstable baseline, access resistance >10% of the membrane resistance).
Replication	Replication was successful in at least 3 independent animals for electrophysiology experiments, 7 independent animals for fiber photometry experiments, and 8 independent animals for behavior experiments. The number of biological replicates reflect the gold standards for the field.
Randomization	The experimental group assignment (control vs stress) was counterbalanced across littermates and age-matched.
Blinding	Investigators were blind to experimental groups whenever possible. The same investigator performed the experimental manipulation (stress vs control) as the electrophysiology experiments, making it challenging to blind during data acquisition.

Reporting for specific materials, systems and methods

We require information from authors about some types of materials, experimental systems and methods used in many studies. Here, indicate whether each material, system or method listed is relevant to your study. If you are not sure if a list item applies to your research, read the appropriate section before selecting a response.

Materials & experimental systems

n/a	Involved in the study
☒	☐ Antibodies
☐	☒ Eukaryotic cell lines
☒	☐ Palaeontology and archaeology
☐	☒ Animals and other organisms
☒	☐ Clinical data
☒	☐ Dual use research of concern
☒	☐ Plants

Methods

n/a	Involved in the study
☒	☐ ChIP-seq
☒	☐ Flow cytometry
☒	☐ MRI-based neuroimaging

Eukaryotic cell lines

Policy information about cell lines and Sex and Gender in Research

Cell line source(s)	HEK293T
Authentication	Purchased from ATCC, stored as low passage numbers (<20)
Mycoplasma contamination	HEK293T cells are routinely tested for mycoplasma contamination using standard PCR approaches.
Commonly misidentified lines (See ICLAC register)	N/A

Animals and other research organisms

Policy information about studies involving animals; ARRIVE guidelines recommended for reporting animal research, and Sex and Gender in Research

Laboratory animals	PV-IRES-Cre: B6;129P2-Pvalb tm1(cre)Arbr/J, Gift from Dr. Diego Restrepo, CU Anschutz (JAX: 008069, RRID:IMSR_JAX:008069)<br>Gt(ROSA)26Sortm9(CAG-tdTomato)Hze (Ai9), The Jackson Laboratory (JAX: 007909, RRID:IMSR_JAX:007909)<br>C57BL/6J, The Jackson Laboratory (JAX: 000664, RRID_JAX:000664)
Wild animals	The study did not involve wild animals.
Reporting on sex	Sex was considered as a biological variable. The sex of the mice was determined by external genitalia. Both male and female mice were used in all experiments and the data are segregated by sex.
Field-collected samples	The study did not involved field-collected samples
Ethics oversight	All procedures were conducted in accordance with guidelines approved by Administrative Panel on Laboratory Animal Care at University of Colorado, Anschutz School of Medicine, accredited by Association for Assessment and Accreditation of Laboratory Animal Care International (AAALAC) (00235). All fiber photometry experiments took place at the University of California, Irvine, and were in accordance with the National Institutes of Health Guidelines for Animal Care and Use.

Note that full information on the approval of the study protocol must also be provided in the manuscript.

Plants

Seed stocks	*Report on the source of all seed stocks or other plant material used. If applicable, state the seed stock centre and catalogue number. If plant specimens were collected from the field, describe the collection location, date and sampling procedures.*
Novel plant genotypes	*Describe the methods by which all novel plant genotypes were produced. This includes those generated by transgenic approaches, gene editing, chemical/radiation-based mutagenesis and hybridization. For transgenic lines, describe the transformation method, the number of independent lines analyzed and the generation upon which experiments were performed. For gene-edited lines, describe the editor used, the endogenous sequence targeted for editing, the targeting guide RNA sequence (if applicable) and how the editor was applied.*
Authentication	*Describe any authentication procedures for each seed stock used or novel genotype generated. Describe any experiments used to assess the effect of a mutation and, where applicable, how potential secondary effects (e.g. second site T-DNA insertions, mosiacism, off-target gene editing) were examined.*
